# Using Machine Learning to Explore Multimodal Digital Markers for Early Detection of Cognitive Impairment in Alzheimer’s Disease

**DOI:** 10.1002/alz.093236

**Published:** 2025-01-09

**Authors:** Joseph Geraci, Edward Searls, Bessi Qorri, Spencer Low, Zexu Li, Philip Joung, Katherine A. Gifford, Abhishek Pratap, Mike Tsay, Christian Cumbaa, Luca Pani, Larry Alphs, Rhoda Au

**Affiliations:** ^1^ NetraMark Corp, Toronto, ON Canada; ^2^ Arthur C. Clarke Center for Human Imagination, School of Physical Sciences, University of California, San Diego, La Jolla, CA USA; ^3^ Department of Pathology and Molecular Medicine, Queen’s University, Kingston, ON Canada; ^4^ Framingham Heart Study, Boston University Chobanian & Avedisian School of Medicine, Boston, MA USA; ^5^ Boston University Chobanian & Avedisian School of Medicine, Boston, MA USA; ^6^ Boston University School of Public Health, Boston, MA USA; ^7^ Framingham Heart Study, Boston University Chobanian & Avedisian School of Medicine, BOSTON, MA USA; ^8^ Vanderbilt Memory & Alzheimer’s Center, Vanderbilt University Medical Center, Nashville, TN USA; ^9^ Boehringer Ingelheim, Ingelheim am Rhein Germany; ^10^ Boston University, Boston, MA USA; ^11^ Department of Psychiatry and Behavioral Sciences, Leonard M. Miller School of Medicine, University of Miami, Coral Gables, FL USA; ^12^ Department of Biomedical, Metabolic, and Neural Sciences, University of Modena and Reggio Emilia, Modena Italy

## Abstract

**Background:**

Recent technological advancements have revolutionized our approach to healthcare, enabling us to harness the potential of smartphones and wearables to collect data that can be used to characterize Alzheimer’s disease (AD) heterogeneity and to develop digital biomarkers. Our focus is to create comprehensive cross‐domain digital datasets and establish an infrastructure that allows for seamless data sharing. Central to accelerating the potential of digital biomarkers for more accurate and early detection is privacy‐protecting data access, which when combined with deep molecular phenotyping, will enhance our understanding of the biological mechanisms underlying clinical expression.

**Methods:**

In the preliminary phase of this project, we analyzed data from 64 participants from the Boston University Alzheimer’s Disease Research Center and encompassing approximately 1480 variables. Our analysis approach leverages a novel machine learning (ML) technology, Attractor AI, that is capable of differentiating causal and non‐causal subpopulations within small patient or study populations and large volumes of measures, enhancing the efficacy of predictive models.

**Results:**

We were able to subcategorize 50% of the 27 cognitively impaired (CI) subjects. A notable discovery was a distinct subpopulation of 8 individuals, 7 of whom were CI, characterized significantly by higher sleep‐derived variables such as various desaturation thresholds and periodicity measures (p=0.008‐0.00007). Additionally, incorporating maximum heart rate, revealed another group of 8 subjects, 6 identified as CI, distinguished by elevated heart rate during one or more of their measuring instances (p=10^‐10^).

**Conclusions:**

While these results are preliminary, they signal a promising direction to cluster subgroups of people along similar dimensions, laying the groundwork for a precision medicine solution. Our future endeavors include expanding the scope of multimodal digital data to encompass aspects like vocalization and speech patterns derived from cognitive assessments, gait analysis, physical activity, and other cognitive tests. The integration of these diverse data streams, coupled with our preliminary sleep analysis findings, has the potential to result in a robust and accurate subgrouping system for CI to help identification of AD risk pathways that might be amenable to early intervention and either delay or prevent potential transition to AD.

